# Changes in Artemin Correlate with Anxiety- and Depression-like Behaviors in a Lipopolysaccharide-Induced Rat Neuroinflammation Model †

**DOI:** 10.3390/biom15081192

**Published:** 2025-08-19

**Authors:** Hasan Çalışkan, Seda Koçak

**Affiliations:** 1 Department of Physiology, School of Medicine, Balıkesir University, Balıkesir 10145, Türkiye; 2 Department of Physiology, School of Medicine, Kırşehir Ahi Evran University, Kırşehir 40100, Türkiye; sdakocak@gmail.com

**Keywords:** artemin, anxiety-like behaviors, depression-like behaviors, neuroinflammation

## Abstract

Artemin is a neurotrophic factor that belongs to the four-member family of Glial-derived growth factors. This study aims to investigate changes in artemin correlated with anxiety and depression-like behaviors in a neuroinflammation rodent model. In adult male Wistar rats, neuroinflammation was established through administration of 2 mg/kg LPS. Anxiety-like behaviors and locomotor activity were evaluated by the open field test. The sucrose preference test and the splash test analyzed depression-like behaviors. Tumor necrosis factor alpha (TNF-*α*), interleukin-1 beta (IL-1β), and artemin levels were measured in the prefrontal cortex, striatum, and serum. In the neuroinflammation group, rearing, total distance traveled, time spent in the central region, and sucrose solution consumption decreased in the open-field test (*p* < 0.0001). Grooming time and frequency were shortened, and grooming latency was prolonged in the neuroinflammation group (*p* < 0.0001). TNF-*α* was significantly increased in the prefrontal cortex (*p* < 0.05) and striatum (*p* < 0.01). lL-1β did not change between groups (*p* > 0.05). Artemin levels decreased in the prefrontal cortex and striatum (*p* < 0.05). No difference was observed in serum artemin levels; however, artemin levels of brain regions were higher than those in the serum. An increase in anxiety–depression-like behaviors has accompanied decreased levels of artemin in the brain. Artemin may be a target molecule in psychiatric disorders. Further studies are needed to examine the role of artemin in neuropsychiatric disorders.

## 1. Introduction

Inflammation is a physiological response in cells and tissues to damage such as injury, infection, and trauma. The inflammatory response eliminates pathogens and initiates tissue-cell healing and angiogenesis [1]. Neuroinflammation can be described as an inflammatory response within the brain or spinal cord [2]. Severe and prolonged neuroinflammation causes damaging effects on neurons and brain functions [3]. Several chemical mediators, such as cytokines, chemokines, and reactive oxygen species, mediate this inflammation. Neuroinflammation can lead to edema, tissue damage, neurodegeneration, microglial priming, cognitive impairments, anxiety, and depression [2]. According to human studies, on serum, cerebrospinal fluid, and postmortem brains, inflammation has been demonstrated to play a role in psychiatric disorders [4,5,6]. Similarly, in preclinical animal studies, both depression- and anxiety-like behaviors were increased in models of inflammation [7,8]. In depression studies, especially despair behavior and anxiety studies, researchers commonly quantify behavioral patterns through duration and frequency measures in aversive regions [9,10]. Neurotrophic factors (NTs) are growth factors with different functions in the nervous system [11]. NTs play a critical role in the nervous system’s development, function, maturation, and survival. These molecules regulate the growth, differentiation, plasticity, stabilization, and survival of neurons [12]. NTs are also involved in the secretion of neurotransmitters from neurons [13]. Further, NTs repair different nervous system defects caused by stress, trauma, and toxic damage [11,14,15]. In particular, impairments in brain-derived neurotrophic factor (BDNF) production in psychiatric diseases have been shown in experimental preclinical studies [16]. Similarly, inflammation has been shown to affect neurotrophic factors negatively [16]. In studies investigating the impact of inflammation on neurotrophic factors, an increase in anxiety and depression-like behaviors has been observed [17]. Cognitive deficits, memory, and memory impairment have also been reported to occur as a result of the inability to function and impaired production of neurotrophic factors [18].

Artemin (ARTN) is termed as neublastin and enovin [19]. ARTN was first identified by Baloh et al. [20]. ARTN belongs to the glial-cell-derived neurotrophic factor family. Other members of this family include glial-cell-derived neurotrophic factor, neurturin, and persephin. ARTN consists of 220 amino acids. The mature form contains 113 amino acids. ARTN is similar in structure and amino acid number to the other three group members. ARTN activates the GFRα3/RET receptor complex. ARTN binds to GFRα3, which is anchored in the cell membrane. By affecting different intracellular signaling pathways, ARTN elicits various effects [21]. ARTN has shown neuroprotective effects in models of spinal cord injury, neuropathic pain, Parkinson’s disease, and toxicity affecting the central nervous system [22,23,24,25].

This study aimed to examine the levels of artemin, a neurotrophic factor in the neuroinflammation model, anhedonia, self-care, locomotor activity, and anxiety-like behaviors. For this purpose, ARTN levels in the prefrontal cortex (PFC) and striatum were investigated in correlation with different behavioral patterns, including self-care behavior and anxiety-like behavior, for the first time.

## 2. Materials and Methods

### 2.1. Animals

The study involved twenty 10-week-old male Wistar Albino rats. Subjects were sourced from the Balıkesir University Experimental Animals Laboratory. Approval from the Balıkesir University Animal Experiments Ethics Committee was received on 28 September 2023, with decision number 2023/8-2. Subjects were divided into control (*n* = 10), and neuroinflammation-induced animals (*n* = 10). Subjects were weighed before being divided into cages. The Guide for the Care and Use of Laboratory Animals was the basis for the conduct of all experiments [26].

### 2.2. Experimental Design

The study administered a single dose of lipopolysaccharide (Sigma Aldrich, *Escherichia coli* O111:B4, catalog number: L2630, St. Louis, MO, USA) 2 mg/kg intraperitoneally [16]. Behavioral tests were conducted 6 h after LPS administration, and sacrifice was conducted 24 h later [16]. The control group received 1 mL/kg of physiologic saline.

### 2.3. Bodyweight Change, Mortality, Posture, and Piloerection Analysis

The weight changes in the subjects and mortality ratio were analyzed. Furthermore, LPS-induced inflammation in test subjects can lead to piloerection and abnormal postures that are not observed in healthy rats. One of these is the prostration posture, which is observed in conjunction with increased inflammation in the central nervous system. In this posture, the animal cannot carry its head and is forced to bend it downward. The other is a hunched posture associated with pain and inflammation. Additionally, fever and increased inflammation can lead to piloerection. The presence or absence of these behaviors in the subjects was investigated to verify the LPS model. The significance of the presence of these behaviors was assessed using Fisher’s exact test.

### 2.4. Open Field Test

The open field test is used to assess general locomotor activity and anxiety-like behaviors. We applied the protocol used in our previous study [27]. The rearing behavior (rearing behavior is when the animal stands on two legs and explores the environment. This behavior decreases with increased anxiety), total distance traveled (locomotor activity parameter), and central zone time (the central region is a risky area. Increased anxiety shortens the time spent in this area) were evaluated. The subjects were taken to the quiet behavior laboratory room at least two hours before the behavioral experiments to allow them to adapt. The behavioral experiments were conducted on the same day for subjects in the same group. The experiment was recorded on camera for a duration of five minutes. After the experiment, the test apparatus was wiped with 70% alcohol. All behavior patterns were analyzed blindly.

### 2.5. Splash Test

The splash test assesses self-care and depression-like behaviors. We applied the protocol used in our previous study [28]. The sprayed sucrose solution triggers self-cleaning behavior in the animals. Subjects start grooming themselves to clean themselves. This situation models self-care behavior. Self-care behaviors decrease in depressed subjects. In the splash test, the duration and frequency of grooming decrease with the increase in depression-like behaviors, but the time until the first grooming increases. A 10% (*g*/*w*) sucrose solution was sprayed on the animals’ dorsal. The total grooming time, grooming frequency, and grooming latency were evaluated. Since the experiment duration was 300 s, the grooming latency was accepted as a maximum of 300 s. For subjects that did not groom, the grooming latency duration was accepted as 300 s. The experiment was recorded on camera for a duration of five minutes. All behavior patterns were analyzed blindly.

### 2.6. Sucrose Preference Test

The sucrose preference test is used to investigate anhedonia, a symptom observed in depression. As a result of the increase in depression-like behavior in the subjects, the consumption of sugar water decreases, and subjects drink more tap water. Animals were offered a 1% sucrose solution or tap water, which they could drink from either a bowl placed on the right or a bowl placed on the left side of the shelter cage. To prevent any effects arising from side preference or neophobia, the positions of the bottles were switched after 12 h. The total volume consumed was noted and used to measure the sucrose preference index. The preference index was determined by the following formula: (sucrose solution consumed volume/total consumed volume) × 100 [29].

### 2.7. Animal Euthanasia and Tissue Collection

The subjects were euthanized with a combination of 50 mg/kg ketamine + 10 mg/kg xylazine, and exsanguinated by cardiac puncture. After anesthesia, the subjects’ posture loss and leg withdrawal reflexes were monitored. When the subjects did not respond to the leg withdrawal reflex, blood was first taken from the left ventricle of the heart. Starting with the occipital region of the skull, the brain was removed by breaking it towards the prefrontal cortex. After the whole brain was removed, the olfactory bulb was separated from the prefrontal hemisphere. The prefrontal cortex and striatum were dissected under the guidance of the rat brain atlas [30]. The removal process was completed in less than 2 min on an ice block. The tissue samples were placed in Eppendorf tubes and immediately transferred to −80 degrees. Tissues were homogenized in potassium chloride buffer at a ratio of 1:9 (0.1 g tissue: 0.9 mL, 140 mmol/L) and then centrifuged at 7000 rpm and 4 °C for 5 min. Brain tissues were homogenized in potassium chloride buffer at a ratio of 1:9 (0.1 g tissue: 0.9 mL, 140 mmol/L) and then centrifuged at 7000 rpm and 4 °C for 5 min. Serum was allowed to clot for 20 min at room temperature. Then the serum was centrifuged at 3000 rpm for 20 min at 4 °C. The supernatants were collected without sediment. The serum obtained was placed in an Eppendorf tube and transferred to −80 °C.

### 2.8. ELISA

ELISA kits were used following the manufacturer’s instructions (serotonin (TNF-α (BT Lab, no: E0764Ra, Shanghai, China), IL-1β (BT Lab, no: 0119Ra, Shanghai, China), artemin (BT Lab, no: E3432Ra, Shanghai, China). PFC, serum, and striatum were examined for ARTN and proinflammatory cytokines. The plates were pre-coated with one of the following antibodies: ARTN, TNF-alpha, or IL-1 beta. Each ELISA plate was pre-coated with the specific capture antibody for the target analyte (ARTN, TNF-α, or IL-1β). For instance, the plate has been pre-coated with rat IL-1β antibody. IL-1β present in the sample is added and binds to antibodies coated on the wells. Following this, biotinylated rat IL-1β antibody is added and binds to IL-1β in the sample. Then streptavidin-HRP is added and binds to the biotinylated IL-1β antibody. After incubation, unbound streptavidin-HRP is washed away during a washing step. Substrate solution is then added, and color develops in proportion to the amount of rat IL-1β. The addition of acidic stop solution terminates the reaction, and the absorbance is measured at 450 nm.

### 2.9. Statistical Analysis

Statistical analyses were conducted using the GraphPad Prism 10.5 software (Boston, MA, USA). Behavior and molecular results were evaluated for normal distribution using the Shapiro–Wilk test. Data displaying a normal distribution were analyzed using the Student *t*-test. Data that did not display a normal distribution were analyzed using the Mann–Whitney U test. For data showing a normal distribution, the arithmetic mean ± SEM was given. For data that do not follow a normal distribution, the arithmetic mean ± SEM, median, and interquartile range were provided. Additionally, brain regions and serum ARTN levels were analyzed by one-way ANOVA (since they showed normal distribution). The Tukey test was used as a post hoc test. *p* < 0.05 was considered statistically significant. A relationship between ARTN levels and behavior patterns was tested using Pearson correlation analysis. The r value and *p* value were given.

According to the Resource Equation Method for animal studies, two-group studies require 6–11 animals in each group [27]. Due to LPS toxicity, the number of subjects in each group was set at 10, taking into account potential subject or sample losses. Furthermore, the results of the morphological parameters were analyzed and evaluated using Fisher’s exact test.

## 3. Results

### 3.1. Bodyweight Change, Mortality, Posture, and Piloerection Results

**Figure 1:**
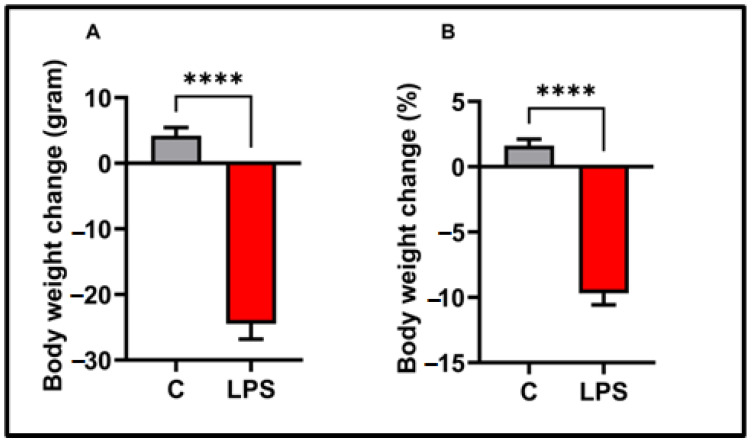
Body weight change findings in the experimental groups, (**A**) body weight change (gram), (**B**) body weight change (%); values in the graphs are presented as the means  ±  SEMs (**** *p*  <  0.0001), (*n* = 10 each group).

According to our study, significant weight loss was observed after LPS application. The results were as follows: LPS group weight loss: 24.50 ± 2.30, median: 27, interquartile range: 3.25, *p* < 0.0001). On the contrary, the control group gained weight (weight gain: 4.20 ± 1.24, median: 3.5, interquartile range: 7.25). The observed weight loss constituted a substantial percentage of body weight (see Figure 1). Compared to the control group, the LPS group lost a significant percentage of body weight (control group weight gain%: 1.63 ± 0.47, median: 1.44, interquartile range: 2.21; LPS group weight loss %: 9.69 ± 0.88, median: 10.34, interquartile range: 1.70). Furthermore, no mortality occurred after LPS administration in the present study (% 0%).

**Figure 2:**
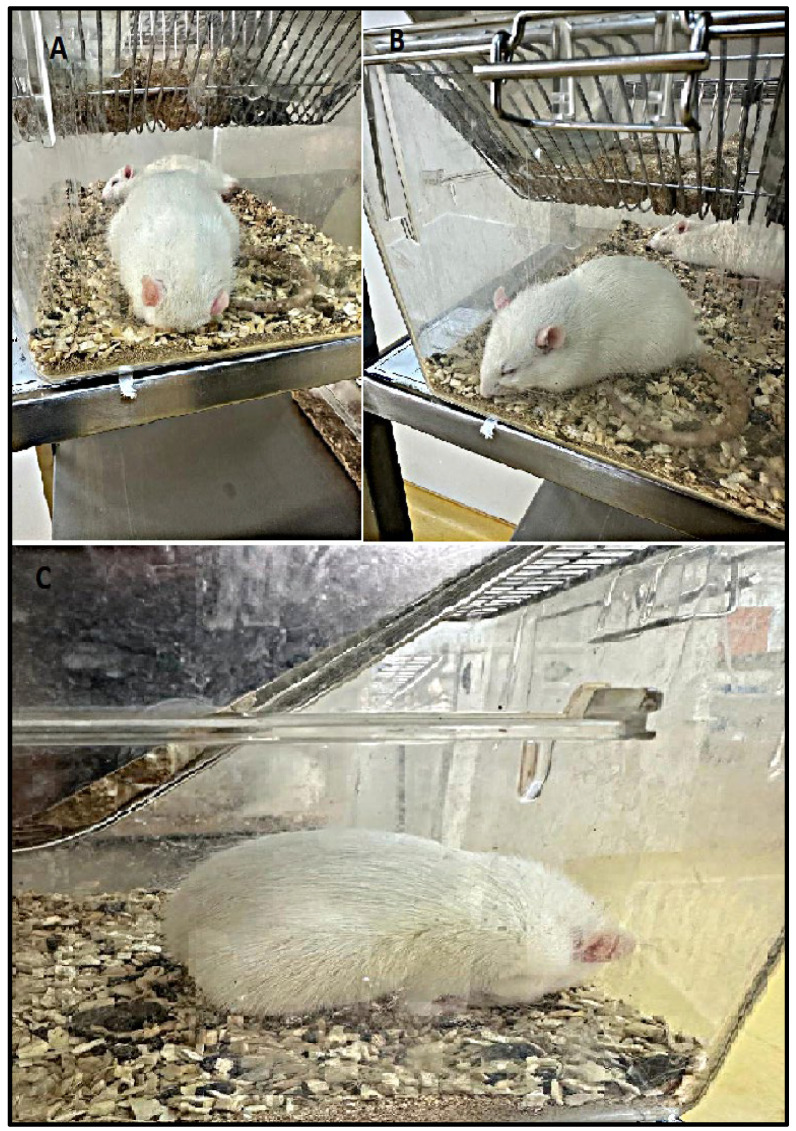
Behavioral findings for the experimental groups: (**A**) prostrate behavior, (**B**) hunched posture, (**C**) piloerection.

Table 1 presents the frequency of morphological parameters observed in male rats from the control and LPS groups, and photos are shown in Figure 2. In the LPS group, prostration posture, hunched posture, and piloerection were observed. These findings were statistically significant compared to the control group. Statistically different from the control group. ✦: *p* = 0.0000108, *p* = 0.000119 (Fisher’s exact test).

### 3.2. Open Field Results

**Figure 3:**
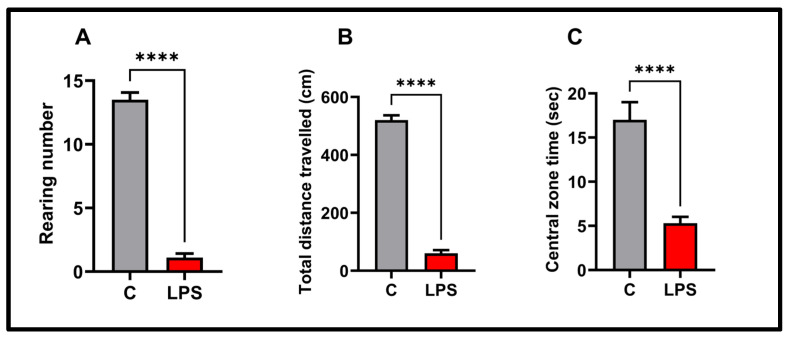
Behavioral findings in the open field test in the experimental groups: (**A**) rearing number, (**B**) total distance traveled, (**C**) central zone time, values in the graphs are presented as the means  ±  SEMs (**** *p*  <  0.0001), (*n* = 10 each group).

We analyzed anxiety-like behaviors and locomotor activity in the open-field test, the results of which are shown in Figure 3. According to the results of the open-field test, the total distance traveled (*p* < 0.0001), rearing number (*p* < 0.0001), and time spent in the center zone (*p* < 0.0001) were significantly decreased in the LPS group compared to the control group.

### 3.3. Splash Test Results

**Figure 4:**
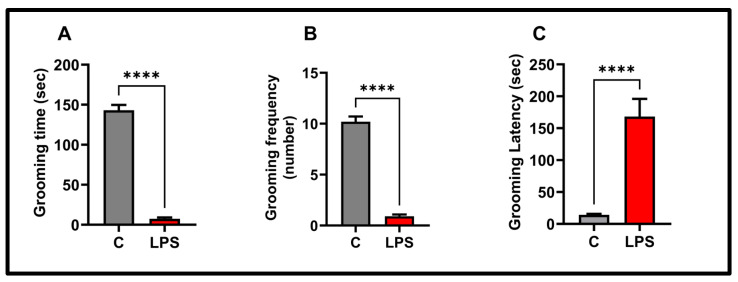
Behavioral findings in the splash test in the experimental groups: (**A**) grooming time, (**B**) grooming frequency, (**C**) grooming latency; values in the graphs are presented as the means  ±  SEMs (**** *p*  <  0.0001), (*n* = 10 each group).

We observed the self-care behavior patterns, the results of which are shown in Figure 4. Grooming time in the splash test (*p* < 0.0001) and grooming frequency (*p* < 0.0001) were significantly reduced in the LPS group. Grooming behavior latency was significantly prolonged in the LPS group (*p* < 0.0001).

### 3.4. Sucrose Preference Test Result

**Figure 5:**
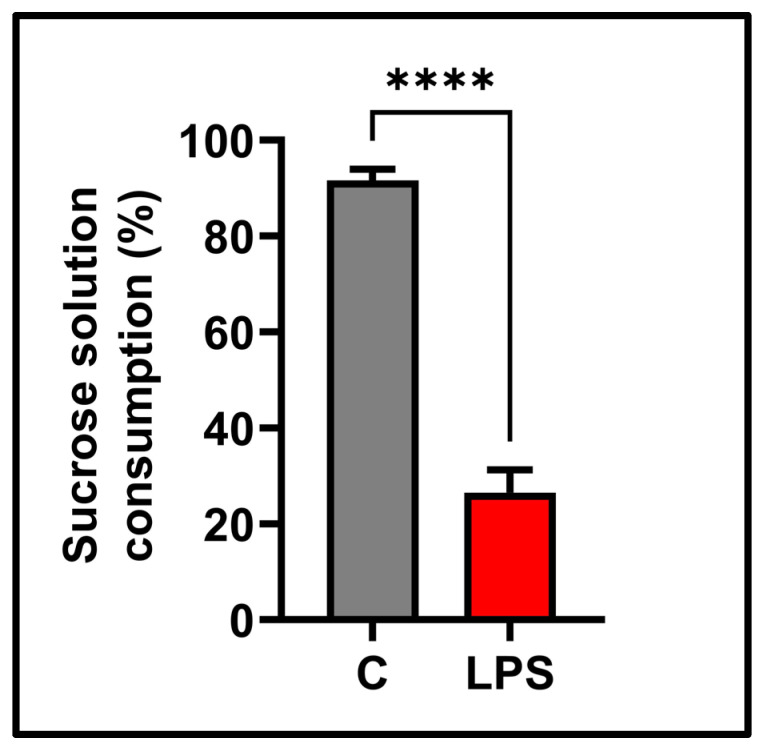
Behavioral finding in the sucrose preference test in the experimental groups, sucrose solution consumption (%); values in the graph are presented as the means  ±  SEMs (**** *p*  <  0.0001), (*n* = 10 each group).

The sucrose choice percentages of the subjects decreased below 65% in the LPS group. Sucrose preference was significantly reduced in the LPS-treated group (*p* < 0.0001). Figure 5 in the sucrose preference test shows the results of anhedonia-like behaviors.

### 3.5. Molecular Results

**Figure 6:**
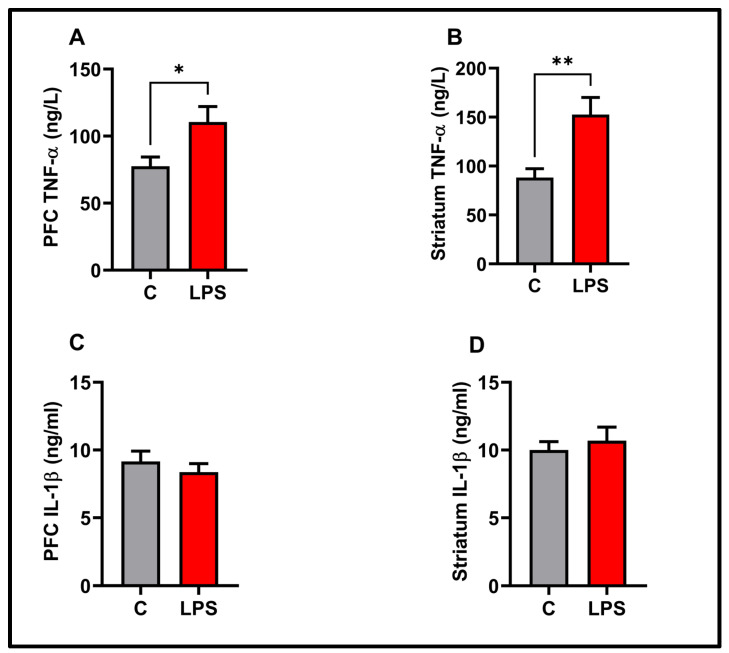
Molecular findings for the experimental groups: (**A**) PFC-TNF-*α*, (**B**) striatum-TNF-*α*, (**C**) PFC-IL-β, (**D**) striatum-IL-1β. Results are presented as mean  ±  SEMs (* *p*  <  0.05, ** *p*  <  0.01), (*n* = 10 each group).

**Figure 7:**
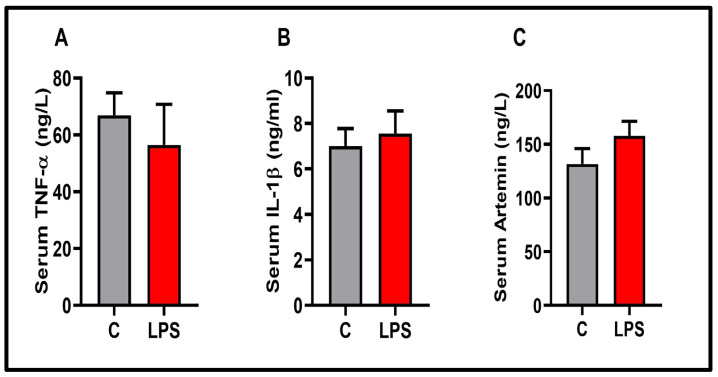
Molecular findings for the experimental groups: (**A**) serum-TNF-*α*, (**B**) serum-IL-1β, (**C**) serum–artemin. Results are presented as mean  ±  SEMs, (*n* = 10 each group).

**Figure 8:**
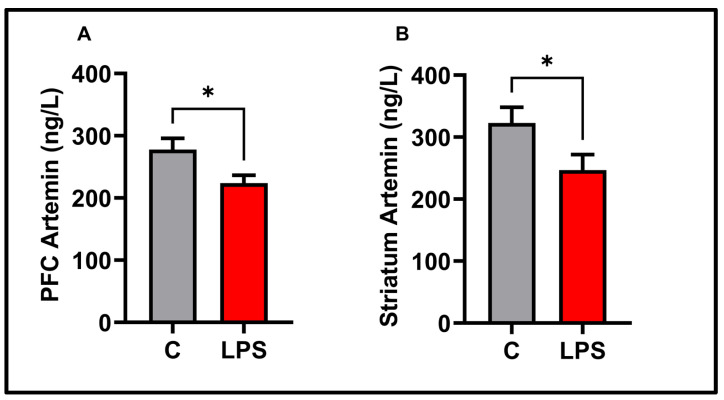
Molecular findings for the experimental groups: (**A**) PFC-artemin, (**B**) striatum–artemin. Results are presented as mean  ±  SEMs (* *p*  <  0.05), (*n* = 10 each group).

**Figure 9:**
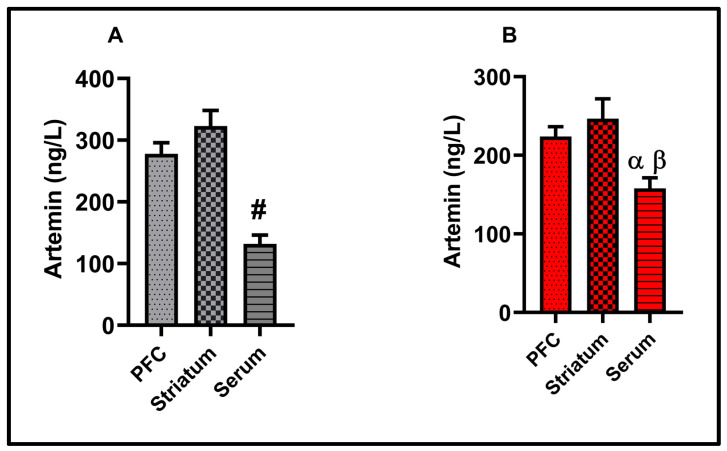
Molecular findings for the Comparison of different brain regions within the same group: (**A**) Control group: PFC-artemin, striatum–artemin, and serum–artemin, (**B**) LPS group: PFC-artemin, striatum–artemin, and serum–artemin. Results are presented as mean  ±  SEMs **#**: *p* < 0.0001 (PFC vs. serum and striatum vs. serum). α: *p*  <  0.05 (PFC vs. serum β: *p* < 0.01 (striatum vs. serum), (*n* = 10 each group).

TNF-*α* and IL-1β data, as proinflammatory cytokines in the present study, are presented in Figure 6. In the prefrontal cortex (*p* < 0.05) and striatum (*p* > 0.01), TNF-*α* was significantly increased. IL-1β slightly increased in striatum, but the increase was insignificant *p* < 0.05). Similarly, no significant change was observed in PFC-IL-1β levels (*p* < 0.05). There was no significant difference in the serum TNF-α, IL-1β, and ARTN levels between the experimental groups (*p* < 0.05) (see Figure 7). ARTN, the neurotrophic factor examined in the present study, is given in Figure 8. ARTN levels were significantly decreased in the prefrontal cortex and striatum (*p* < 0.05). Further, brain regions and serum ARTN levels were analyzed in both the control and LPS groups. No difference was observed between the prefrontal cortex and striatum in the control group (*p* > 0.05). However, ARTN levels were found to be significantly higher in both the prefrontal cortex and striatum compared to serum (*p* < 0.0001) (see Figure 9A). No difference was observed between the PFC and striatum in the LPS group (*p* > 0.05) (see Figure 9A). Similarly, higher ARTN levels were measured in the PFC and striatum compared to serum in the LPS group (PFC vs. serum *p* < 0.05, striatum vs. serum: *p* < 0.01), (see Figure 9B).

### 3.6. Correlation Results

**Figure 10:**
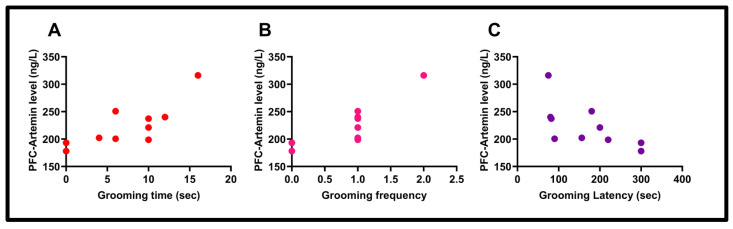
The correlation between PFC artemin level and grooming time (r = 0.78), (*p* = 0.0068) (**A**), the correlation PFC artemin level and grooming frequency (r = 0.82) (**B**), (*p* = 0.0032), and the correlation between PFC artemin level and grooming latency (r = −0.64), (*p* = 0.0445) (**C**).

In the neuroinflammation group, a statistically significant correlation was found between PFC ARTN levels and grooming time (“Pearson Correlation”, r  =  0.78, *p*  =  0.0068). Similarly, in the neuroinflammation group, a statistically significant correlation was found between PFC ARTN levels and grooming frequency (“Pearson Correlation”, r  =  0.82, *p*  =  0.0032). A remarkable correlation was also observed between PFC-ARTN levels and grooming latency (“Pearson Correlation”, r  = −0.64, *p*  =  0.0445) (see Figure 10).

**Figure 11:**
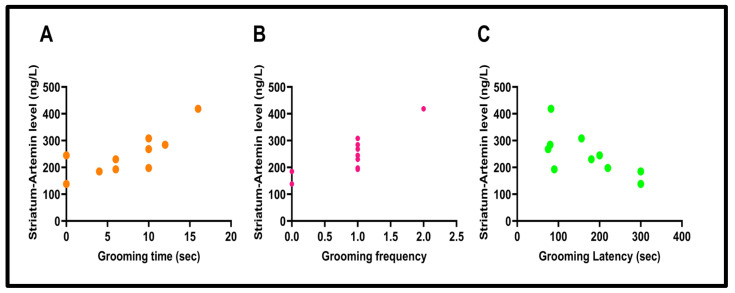
The correlation between striatum–artemin level and grooming time (r = 0.77) (**A**), (*p* = 0.0081), the correlation striatum–artemin level and grooming frequency (r = 0.84), (*p* = 0.0020) (**B**), and the correlation between striatum–artemin level and grooming latency (r = − 0.67), (*p* = 0.0330) (**C**).

The correlation between striatal ARTN levels and grooming time (“Pearson Correlation”, r  =  0.77, *p*  =  0.0081), grooming frequency (“Pearson Correlation”, r  =  0.84, *p*  =  0.0020), and grooming latency (“Pearson Correlation”, r  =  −0.67, *p*  =  0.0330) was investigated and found to be significant in all three correlations (see Figure 11).

**Figure 12:**
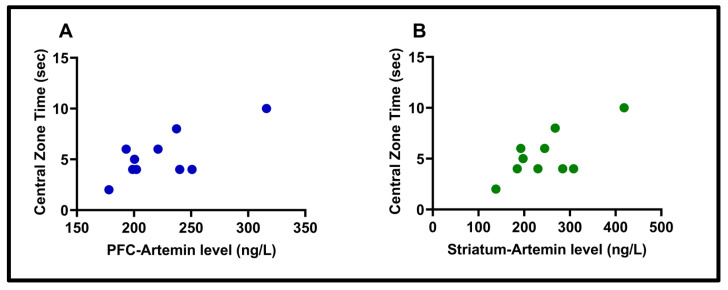
The correlation between PFC artemin level and central zone time (r = 0.73), (*p* = 0.0159) (**A**); the correlation between striatum–artemin level and central zone time (r = 0.71), (*p* = 0.0209) (**B**).

In the open field test, a significant correlation was found between the time spent in the central area and the levels of ARTN in both the striatum (“Pearson Correlation”, r  =  0.71, *p*  =  0.0209) and PFC (“Pearson Correlation”, r  =  0.73, *p*  =  0.0159) (see Figure 12).

## 4. Discussion

In the present study, a neuroinflammation model with lipopolysaccharide was established. Significant behavioral and molecular changes were observed.

In the open field test, vertical (rearing behavior) and horizontal locomotor activity (total distance traveled) decreased. Neuroinflammation caused hypolocomotion. After LPS administration, different studies reported reduced locomotor activity in mice and rats [31,32]. In the present study, anxiety-like behaviors increased in the subjects. The subjects spent time in compartments close to the walls of the open field test. This anxiety-related thigmotaxis was observed in all LPS-treated subjects. Other researchers have reported similar behavioral observations [16,31,32]. Furthermore, anxiety-like behaviors were also reported to increase in the elevated plus maze and light–dark box tests, which are unconditioned anxiety tests, as a result of neuroinflammation [16].

In experimental depression studies, anhedonia, behavioral despair, and decreased self-care behaviors are studied with different models [27,28]. Behavioral despair has also been reported; after LPS administration, especially in the forced swimming test of rodents, depression-like behaviors increase [7].

Similarly to the forced swimming test, there are consistent results in the tail suspension test, which is usually applied to mice [10]. Generally, LPS studies focus on anhedonia and behavioral despair in depression. In contrast to other LPS studies, the present study focused specifically on self-care behaviors. In the present study, pollution was stimulated with a high-density sucrose solution. While healthy rats in the control group exhibited grooming behavior quickly, grooming behavior was significantly reduced in the LPS-treated group. In depression, the time and frequency of self-care behaviors are seriously affected. In the present study, grooming behavior patterns, a crucial self-care behavior, were disrupted in rats after LPS administration. The study also observed that the subjects decreased their consumption of sucrose water and exhibited anhedonic behaviors.

In neuroinflammation models, anhedonia and behavioral despair are associated with increased proinflammatory cytokines [7,10]. In this study, a self-care deficit was identified in conjunction with these behavioral patterns.

To confirm the model, proinflammatory cytokines TNF-*α* and IL-1β were analyzed. An increase in TNF-*α* was observed in both brain regions examined. A non-significant moderate increase in IL-1β was observed. In studies with LPS, a dampening of the levels of some cytokines was observed [33,34]. This may be related to the route of LPS administration, tissue examined, age and sex of the animal, LPS dose, and sacrifice time [16,33,34].

In the present study, LPS administration was performed at noon. The subjects were sacrificed 24 h after the experiment. Some misleading results may be obtained, especially in rats administered in the morning and sacrificed 24 h later. High glucocorticoid levels in the morning may cause a dampening of cytokines in rats. Therefore, the administration of injections and the time of sacrifice are essential [16]. Postural changes and piloerection observed after exposure to different infections have been investigated in the LPS model [35,36]. Prostration, hunched posture, and piloerection were observed in the neuroinflammation group. These findings may be helpful in both establishing the LPS model and in examining the conditions of the animals before sacrifice. Further, post-LPS treatment approaches and improvements in piloerection and postures can also be analyzed. Furthermore, weight loss and mortality may be observed after LPS administration [16]. Significant weight loss was observed in the presented study. No mortality occurred within the first 24 h. Similarly to our previous study data, subjects lost weight [16]. Previously, we observed a 20% mortality rate in 16-week-old male Wistar rats after a similar 2 mg/kg LPS administration [16]. In the present study, we did not observe any mortality in 10-week-old male Wistar rats. Age may be an essential factor in neuroinflammation models induced by LPS.

ARTN is a growth factor of glial origin. There is a minimal number of studies on the physiological functions of ARTN. ARTN has been shown to have physiological functions in maintaining the survival of peripheral ganglia and dopaminergic neurons [20]. ARTN helps regulate the differentiation of autonomic, sensorimotor, and enteric neurons in the central and peripheral nervous systems [37]. Further, ARTN improves neuronal survival, proliferation, and regeneration [20]. ARTN takes part in the process of initiating migration and axon projection from sympathetic neurons [38].

Psychiatric studies on ARTN are related to depression and anxiety. Patients with major depression had low levels of ARTN in serum [39]. In patients diagnosed with generalized anxiety disorder who did not receive drug treatment and did not have depression, artemin mRNA levels in serum were found to be higher than those of healthy controls [40]. In ulcerative colitis patients with depression and anxiety, artemin was not associated with the abdominal pain process [41].

Two preclinical studies are reporting different findings regarding ARTN [42,43]. In the LPS-induced depression model, artemin levels in the prefrontal cortex were found to be high after 24 h in male mice, while BDNF levels did not change [42]. These results, which were also found interesting by the study team, may be related to the type and dose of LPS used, as well as the animal species. After LPS administration, artemin levels decreased in the PFC in the present study. BDNF levels also decreased in another study in male rats, where we administered LPS treatment [16]. In another experimental study, artemin levels increased in the spinal cord, PFC, and hippocampus, and depression-like behaviors decreased in parallel [43]. These findings are consistent with the decrease in ARTN levels and increase in depression–anxiety behaviors after LPS administration in our study.

Studies on the striatum and artemin underscore the importance of artemin, particularly concerning dopaminergic neurons. In female Sprague Dawley rats with striatal lesions induced by 6-hydroxydopamine, ARTN was adversely affected [44]. Following methamphetamine toxicity, a decrease in dopamine levels in the striatum was observed, and the exogenous administration of mitigated this decrease [45]. According to another preclinical study, following the lesion created with 6-hydroxydopamine, the application of ARTN ensured that approximately four times more dopaminergic neurons survived [23]. In another preclinical study, the combination of artemin and heparin demonstrated good distribution in the striatum [46]. In the presented study, LPS was found to downregulate artemin levels in the striatum. In neurodegenerative diseases characterized by high inflammation, ARTN appears to be both a potential therapeutic agent and an essential neurotrophic factor, shedding light on the pathophysiological processes.

In our study, we examined the relationship between artemin and behavioral patterns in detail using correlation analysis. Sucrose water causes dirtiness and stickiness in the fur of rats. The rats’ cleaning of their fur to remove the dirt represents self-grooming behavior [28]. Decreased artemin levels in the prefrontal cortex and striatum negatively affected self-grooming behavior. As ARTN levels decreased in both brain regions, grooming time and frequency also reduced. This significant correlation may be an essential finding for translational medicine regarding artemin’s role in disrupted self-grooming behaviors associated with depression. Furthermore, control groups began to clean themselves quickly after being sprayed with sugar water. In the LPS group, grooming latency increased with a decrease in brain ARTN levels. As ARTN levels decreased, the first self-grooming behavior was delayed. A significant correlation was observed between these two findings. In summary, the decrease in artemin also led to a delay in the onset of self-care behavior.

In addition, as artemin levels decreased in both the PFC and striatum, the time spent in the center of the open-field test decreased. The significant correlation observed here is that a decrease in artemin levels is associated with an increase in anxiety-like behavior. As ARTN levels decreased, subjects exhibited thigmotaxis, moving toward the periphery of the open-field test.

The PFC and the striatum interact in a crucial way to support cognitive processes [47]. The information from the PFC is processed in the striatum, which is a structure in the brain. Anxiety and depression can be caused by disruptions in the processing of both regions [48,49]. Brain-derived neurotrophic factors were shown to be transported from the PFC to the striatum by anterograde transport [50]. The expression of the BDNF gene in the PFC may also affect other brain regions, such as the striatum. Both brain regions exhibited comparable levels of artemin, as revealed by our findings. The exact process may be responsible for the transportation of ARTN from the PFC to the striatum, namely anterograde transport, in a manner analogous to that of BDNF. The present study found no significant difference between the PFC and striatum regions. Furthermore, these two regions were similarly affected by LPS. The absence of interregional differences and the presence of similar levels of adverse effects in the control group suggest that artemin expression may be transferred between regions.

Several hypotheses exist regarding whether LPS, when applied peripherally, crosses the blood–brain barrier and how it induces inflammation and microglial activation. Some authors have reported that LPS does not cross the blood–brain barrier, instead activating microglia indirectly [51,52]. This activation may be mediated by endothelial cells in the blood–brain barrier [52], changes in the permeability of the blood–brain barrier caused by LPS [53] or stimulation of the vagus nerve or other afferent nerves [54,55]. Banks and Robinson reported that, in male CD-1 mice, LPS labeled with a radioactive atom crosses the blood–brain barrier in very low amounts [56]. Vargas-Caraveo et al. reported that LPS crosses the blood–brain barrier via lipoprotein-mediated transport in the male Wistar Hannover rats [57]. LPS activates the Toll-like receptor 4, triggering the release of proinflammatory cytokines, reactive oxygen species, and various chemical mediators [58]. This receptor is found in many different types of cells, including microglia in the central nervous system [58]. An increase in proinflammatory cytokines, reactive oxygen species (ROS), and other chemical mediators can disrupt the brain’s chemical balance, including neurotransmitters, neurotrophic factors, and glucocorticoids [59,60,61]. This chemical imbalance leads to negative behavioral changes [16].

LPS can cause a high cytokine storm, as well as other effects. LPS disrupts the permeability of the blood–brain barrier, increases oxidative stress, and disrupts the hypothalamic–pituitary–adrenal axis [62,63,64]. The disrupted blood–brain barrier, increased oxidative stress, and hypothalamic–pituitary–adrenal axis dysregulation may also have negatively affected ARTN expression. Liang et al. found that exogenous artemin administration reduced inflammatory responses and nitric oxide production in mice [65].

LPS also disrupts the chemical balance between excitatory and inhibitory factors [66].

Glutamate is the common excitatory neurotransmitter in the central nervous system, and its toxicity causes many problems [67,68]. Some authors consider inflammation, glutamate toxicity, and glial cell dysfunction to be the triad causing problems in mood disorders [69]. The investigation of the relationship between artemin and glutamate may also be useful for possible therapeutic and pathological mechanisms.

Serum samples may be necessary from a translational medicine perspective. In the presented study, no significant change was observed in serum artemin levels. However, the presented artemin levels were found to be significantly higher in the central nervous system compared to serum. Pallanti and colleagues found ARTN levels to be high in the group of patients with generalized anxiety disorder and significantly lower in patients with depression [40]. In addition to the data presented in this study, further investigation of artemin may be beneficial in diseases characterized by high levels of neuroinflammation. A high prevalence of anxiety and depression has been found in neuroinflammatory diseases [70,71]. Our results revealed high levels of artemin in the central nervous systems of both diseased and healthy rats compared to serum levels, suggesting that examining cerebrospinal fluid in addition to serum may be valuable in clinical settings for neuroinflammated patient groups.

The study has some limitations. Body temperature changes that may occur after LPS administration have not been measured, as they may affect behavior. It may be helpful to measure body temperature, heart rate, and blood pressure using advanced techniques without affecting behavior. In models where LPS is administered systemically, the duration of the experiment is usually short [72,73]. This duration is a limitation in terms of disease patterns in humans. The examination of behaviors in a chronic neuroinflammation model with intracerebral ventricular and lower doses of LPS administration may be useful. Due to the study budget, only male rats were used in the experiment. We believe that the study should also be conducted with female rats. The subjects’ body temperature changed after LPS administration. Increased body temperature may have affected artemin production. Body temperature was not measured because it may affect the animals’ behavior.

## 5. Conclusions

In the neuroinflammation model, the levels of artemin in the prefrontal cortex and striatum of the subjects decreased. Subjects’ frequency and duration of self-care-related grooming behavior decreased. Subjects’ anhedonia and anxiety-like behaviors increased. A strong correlation was found between reduced levels of artemin in both the prefrontal cortex and striatum and impaired self-care behavior parameters. Further studies on artemin are needed in terms of different brain regions and receptors.

## Figures and Tables

**Table 1:** The frequency of morphology observed in male rats from the control and LPS groups.

	Control Group	LPS Group
Prostration posture	0/10	9/10 **^✦^**
Piloerection	0/10	10/10 **^#^**
Hunched posture	0/10	10/10 **^#^**

^✦,#^ Statistically different from the control group. ^✦^: *p* = 0.0000108, *p* = 0.000119 (Fisher’s exact test).

## Data Availability

The data supporting this study’s findings are available from the corresponding author upon reasonable request.

## Abbreviations

The following abbreviations are used in this manuscript:

ARTN	Artemin
BDNF	Brain-derived neurotrophic factor
IL-1β	Interleukin-1beta
LPS	Lipopolysaccharide
NTs	Neurotrophic factors
PFC	Prefrontal cortex
TNF-*α*	Tumor necrosis factor-alpha
